# Reduction of the geomagnetic field delays *Arabidopsis thaliana* flowering time through downregulation of flowering‐related genes

**DOI:** 10.1002/bem.22123

**Published:** 2018-04-30

**Authors:** Chiara Agliassa, Ravishankar Narayana, Cinzia M. Bertea, Christopher T. Rodgers, Massimo E. Maffei

**Affiliations:** ^1^ Plant Physiology Unit Department of Life Sciences and Systems Biology University of Turin Turin Italy; ^2^ Department of Entomology University Park Pennsylvania; ^3^ The Wolfson Brain Imaging Centre Cambridge Biomedical Campus Cambridge United Kingdom

**Keywords:** near‐null magnetic field, geomagnetic field, *Arabidopsis thaliana*, leaves and floral meristem gene expression, delay in flowering time

## Abstract

Variations in magnetic field (MF) intensity are known to induce plant morphological and gene expression changes. In *Arabidopsis thaliana* Col‐0, near‐null magnetic field (NNMF, i.e., <100 nT MF) causes a delay in the transition to flowering, but the expression of genes involved in this response has been poorly studied. Here, we showed a time‐course quantitative analysis of the expression of both leaf (including clock genes, photoperiod pathway, *GA20ox*, *SVP*, and vernalization pathway) and floral meristem (including *GA2ox*, *SOC1*, *AGL24*, *LFY*, AP1, *FD*, and *FLC*) genes involved in the transition to flowering in *A. thaliana* under NNMF. NNMF induced a delayed flowering time and a significant reduction of leaf area index and flowering stem length, with respect to controls under geomagnetic field. Generation experiments (F_1_‐ and F_2_‐NNMF) showed retention of flowering delay. The quantitative expression (qPCR) of some *A. thaliana* genes expressed in leaves and floral meristem was studied during transition to flowering. In leaves and flowering meristem, NNMF caused an early downregulation of clock, photoperiod, gibberellin, and vernalization pathways and a later downregulation of *TSF*, *AP1*, and *FLC*. In the floral meristem, the downregulation of *AP1*, *AGL24*, *FT*, and *FLC* in early phases of floral development was accompanied by a downregulation of the gibberellin pathway. The progressive upregulation of *AGL24* and *AP1* was also correlated to the delayed flowering by NNMF. The flowering delay is associated with the strong downregulation of *FT, FLC*, and *GA20ox* in the floral meristem and *FT*, *TSF*, *FLC*, and *GA20ox* in leaves. Bioelectromagnetics. 39:361–374, 2018. © 2018 The Authors. *Bioelectromagnetics* Published by Wiley Periodicals, Inc.

## INTRODUCTION

The Earth's magnetic field (MF), also known as the geomagnetic field (GMF), is an environmental factor affecting all organisms living on the planet, including plants. The GMF protects the Earth and its biosphere from the lethal effects of solar wind by deflecting most of its charged particles through the magnetosphere away into space [Occhipinti et al., 2014].

Since plants respond to environmental stimuli such as light and gravity with so‐called phototropic and gravitropic responses, it is no wonder that the GMF is also able to influence many biological processes in plants [Maffei, 2014]. In recent years, the progress and status of research on the effect of MFs on plants has been reviewed [Phirke et al., 1996; Abe et al., 1997; Belyavskaya, 2004; Galland and Pazur, 2005; Minorsky, 2007]. The effects of both weak and strong MFs have been thoroughly discussed, with a particular focus on the involvement of GMF reversal events on plant evolution [Maffei, 2014]. However, a detailed analysis of experiments describing the effects of MFs on plants shows a large number of conflicting reports, characterized by a dearth of plausible biophysical interaction mechanisms. Many experiments are simply unrealistic, while others lack a testable hypothesis and, ultimately, prove not to be reproducible [Harris et al., 2009].

A large number of studies on MF effects on plants have been carried out by using MF intensity higher than the GMF; however, only a limited number of studies have analyzed the effects of exposure of plant to MF with intensity lower than the GMF [Maffei, 2014]. The term “weak” or “low magnetic field” generally refers to intensities from 100 nT to 0.5 mT, whereas “super‐weak,” “conditionally zero,” or “near‐null magnetic field” (NNMF) refers to MFs below 100 nT [Maffei, 2014].

Investigations of NNMF effects on biological systems have attracted the attention of biologists for several reasons. Reversal of the GMF implies a period of transition that may expose living organisms to NNMF. Besides the described effects of GMF reversals and their effects on plant evolution [Occhipinti et al., 2014], interplanetary navigation will introduce humans, animals, and plants to environments where the natural MF is near 1 nT, unless artificially augmented. Therefore, the topic is of wide interest.

In *Arabidopsis thaliana* seedlings grown under NNMF, preliminary results showed that flowering time was found to be delayed compared with seedlings grown in normal GMF [Xu et al., 2015, 2017, 2013, 2018]. Moreover, the transcription level of a few flowering‐related genes also changed [Xu et al., 2012]. Furthermore, the biomass accumulation of plants in NNMF was significantly suppressed at the time when plants were switching from vegetative growth to reproductive growth compared to that of plants grown in normal GMF. This was caused by a delay in flowering of plants in NNMF, which resulted in a significant reduction in the harvest index of plants in NNMF compared with that of control plants. Therefore, preliminary results indicate that the removal of the local GMF negatively affects the reproductive growth of *A. thaliana*, which thus affects the yield and harvest index [Xu et al., 2013]. Since timing of flowering is crucial to the life cycle of plants, it is not surprising that plants constantly monitor environmental signals to adjust the timing of the floral transition [Capovilla et al., 2015], but it is amazing that this is exquisitely sensitive to MF.

**Figure 1:**
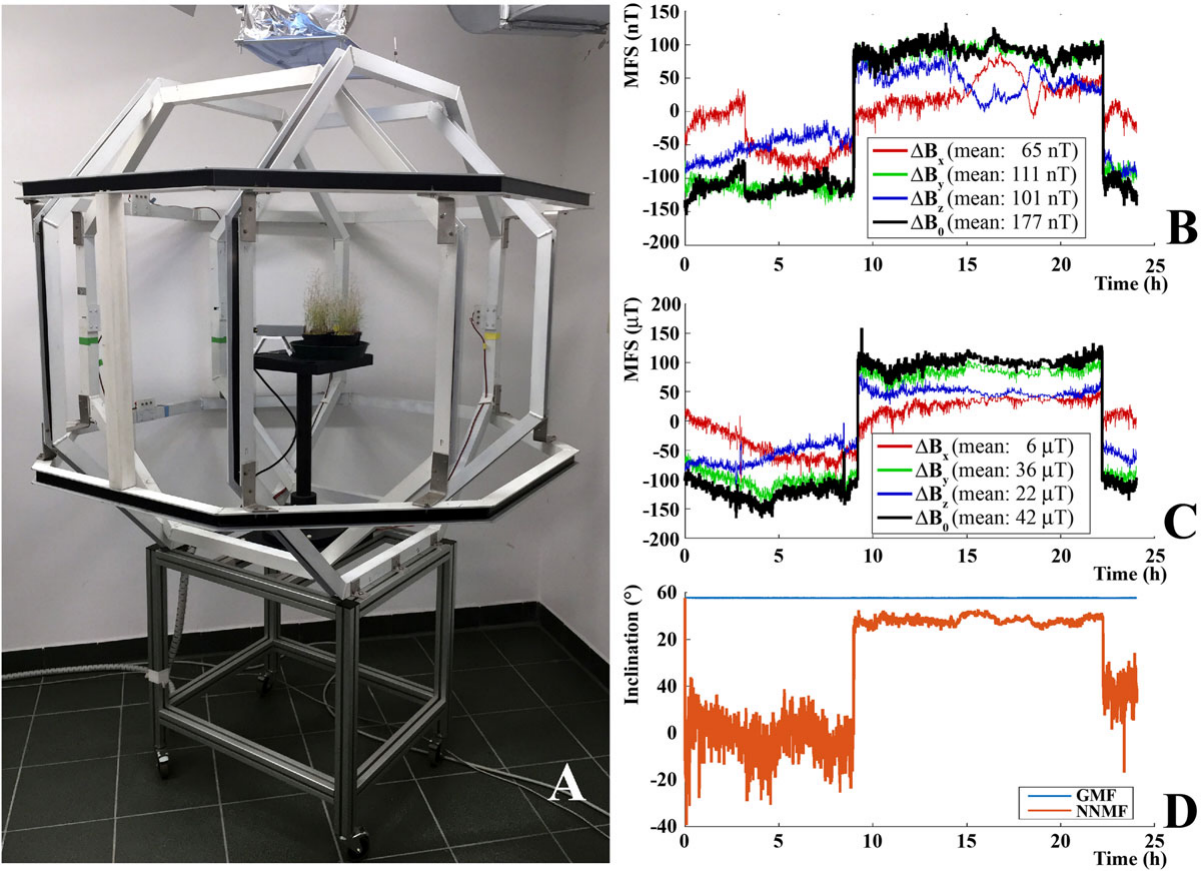
Geomagnetic field compensation system. (**A**) Triaxial coils (comprised of a Helmholtz pair of octagonal coils for each of three perpendicular axes) for cancelling the geomagnetic field. (**B**) Example of a plot of residual MF measured by Bartington Fluxgate magnetometer during a near‐null MF experiment. (**C**) Same measurements, but during GMF. (**D**) Expected variations of magnetic inclination under NNMF. Inclination was defined as $\text{Arctan}\left(\frac{B_{y}}{\sqrt{Bx^{2}+Bz^{2}}}\right)$. The *y*‐axis was defined to run vertically, and *x*‐ and *z*‐axes were horizontal. MFS, magnetic field strength (mean values).

While the effects of day length (photoperiod) [Sanchez et al., 2011] and temperature changes [Chew et al., 2012] on flowering time have been thoroughly studied, many aspects of plant flowering delay in response to NNMF are still poorly explored. Plant flowering time is controlled by several genes, including circadian clock‐associated genes [Hara et al., 2014], genes involved both in the transition from the vegetative to the reproductive phase [Gu et al., 2013] and in the precise control of flowering [Song et al., 2014], and microRNA regulation [Spanudakis and Jackson, 2014; Hong and Jackson, 2015]. Current models provide us with a basis on which to address a number of fundamental issues for a better understanding of the molecular mechanisms by which plants respond to environmental stimuli to control flowering time [Fornara et al., 2010]. Therefore, to assess the effect of NNMF on *A. thaliana* flowering time, we built an MF compensation apparatus, comprised of three orthogonal Helmholtz coil pairs under computer control, of dimensions sufficient for plants to grow from seed to seed. This apparatus is able to accurately reduce the normal GMF to NNMF (ca. 40 nT) (Fig. 1). This apparatus was also instrumental in our assessment of the effect of GMF reversal on *A. thaliana* gene expression [Bertea et al., 2015].

Previous work has shown that flowering time is delayed and the expression of a few *A. thaliana* genes involved in the transition to flowering is altered after exposure to NNMF [Xu et al., 2012]. However, the limited number of flowering genes analyzed in this study (*COP1*, *CO*, *FT*) and the lack of analysis of organ‐specific gene expression do not allow for evaluation of either the interaction between genes expressed in the leaves and floral meristem or the dynamics of modulation of gene expression. In this work, we used the authoritative “SnapShot” atlas [Fornara et al., 2010] to select the key genes from all major pathways responsible for the control of flowering, expressed in the leaves and also in the floral meristem, and we significantly extended the preliminary observations of previous works [Xu et al., 2012, 2013]. For the first time, we were also able to make a comprehensive, time‐course, Real‐Time PCR analysis of NNMF effects.

We hypothesized that exposure of *A. thaliana* to NNMF would affect flowering pathways to different extents and that understanding which pathways are most sensitive to NNMF would give clues to the mechanism of plant magnetoreception.

## MATERIALS AND METHODS

### Plant Material and Growth Conditions

*A. thaliana* ecotype Columbia 0 (Col‐0) wild type seeds were sown in 8 cm diameter polyethylene pots with soil prepared with a mixture of peat and vermiculite (2:1). Sown pots were exposed to homogenous irradiation from a high pressure sodium lamp source (Grolux 600W, Sylvania, Wilmington, MA) at 200 μmol m^−2^ s^−1^, at 21 °C (±1.5 °C) with a photoperiod of 14 h light and 10 h darkness. Control plants were exposed to normal Earth magnetic field (GMF), in the same laboratory and at the same time, under controlled light and temperature identical to those in the triaxial coils. Control experiments (GMF) were performed in the same lab at a distance of 8 m from the triaxial Helmholtz coils, and the measured levels of power‐line frequency (50 Hz) MF associated with the triaxial coils and control GMF were similar. Treated plants were grown inside the triaxial coils under NNMF (see below in the section NNMF Generation System).

Seeds from plants growing either in the GMF control or under NNMF were harvested from brown siliques which were carefully cut at their base. In order to evaluate the generation effect, seeds were sieved to separate them from chaff and were kept in small Petri dishes (4 cm diameter) and maintained under either GMF or NNMF for 2 weeks. These seeds were then sown in pots as described above. The seeds of the first experiment (F_1_ NNMF and F_1_ GMF seeds) were collected and sown in pots as described above and plants were allowed to grow until full bloom. Seeds of F_1_ NNMF and F_1_ GMF (defined as F_2_ NNMF and F_2_ GMF) were then collected as above and kept under either GMF or NNMF for 2 weeks. These seeds were sown in pots as described above in order to obtain a third generation of plants experiencing either NNMF or GMF.

Leaf area index (LAI) was measured by dividing the leaf area by the pot area. Stem length was measured from the base to the tip of the flowering stem. Pictures were taken for all generations and the phenotypic behavior (leaf area index and stem length) was plotted as a function of time.

### NNMF Generation System

The GMF (or local geomagnetic field) values were typical of the Northern hemisphere at 45°0′59″ N and 7°36′58″ E coordinates. Near‐null MF was generated by three orthogonal Helmholtz coils (Fig. 1A) connected to three DC power supplies (model E3642A 50W, 2.5Adual range: 0‐8V/5A and 0‐20V/2.5A, 50W, Agilent Technologies, Santa Clara, CA) controlled from a computer via a GPIB connection. A real‐time measure of the MF in the plant exposure chamber was achieved with a three‐axis MF sensor (model Mag‐03, Bartington Instruments, Oxford, UK) that was placed at the geometric center of the Helmholtz coils. The output data from the magnetometer were uploaded to VEE software (Agilent Technologies) to fine‐tune the current applied through each of the Helmholtz coil pairs in order to maintain the MF inside the plant growth chamber at NNMF intensity. Defining the vertical axis as “*y*,” the GMF level at the experimental location in our lab was *B_x_* = 6.39 μT, *B_y_* = 36.08 μT, *B_z_* = 20.40 μT; i.e., an MF strength (*B* = [*Bx* ^2^ + *By* ^2^ + *Bz* ^2^]^1/2^) of 41.94 μT; by applying the following voltages *Vx* = 11.36, *Vy* = 15.04, *Vz* = 13.81 (which produced currents *I_x_* = 26 mA, *I_y_* = 188 mA, *I_z_* = 103 mA), the magnetometer values were *B_x_* = 0.033 μT, *B_y_* = 0.014 μT, *B_z_* = 0.018 μT with a field strength of 40.11 nT, which is about one thousandth of the GMF strength (Fig. 1B and C). The coil diameter (*Ø*) and separations between the Helmholtz coils (sep.) were the following: *X*, *Ø* = 128 cm, sep. = 55 cm; *Y*, *Ø* = 150, sep. = 67; *Z*, *Ø* = 135, sep. = 59 (Fig. 1A). The inclination angle of B in GMF was 57.7 degrees, and in NNMF the inclination of the tiny residual field B varied between −71.3 and +44.5 degrees (see Fig. 1D). Supplementary Figure S1 shows main field inclination values on a world scale.

Sham exposure experiments were performed by keeping the field almost identical to that of the GMF but altering the direction (i.e., declination, or “North, East, or West”) of the horizontal component of the field with equal currents in the triaxial coils compared to the NNMF (see above) by altering the voltage of the coils. This sham exposure ruled out potential subtle heating or vibrational effects either from the coils themselves or from the electronics used to control the coils. Sham experiments resulted in insignificant differences between GMF and altered inclination of the GMF (data not shown). Because GMF are the natural conditions experienced by plants, we chose to use GMF as control.

Double‐blind experiments were performed by applying field blinded from the personnel performing the remainder of the experiments and/or interpreting the data.

### RNA Isolation from Plants Grown Under Either NNMF or GMF

Since the expression levels of flowering genes vary as a function of time of day, we chose to collect leaves and floral meristems from plants growing either under GMF (control) or NNMF (treatment) at noon (12:00). Samples were immediately frozen in liquid nitrogen. Fifty milligrams of either frozen leaf or frozen floral meristem material were ground in liquid nitrogen with mortar and pestle. Total RNA was isolated using Agilent Plant RNA Isolation Mini Kit (Agilent Technologies) and RNase‐Free DNase set (Qiagen, Hilden, Germany). Sample quality and quantity were checked using RNA 6000 Nano kit and Agilent 2100 Bioanalyzer (Agilent Technologies), following the manufacturer's instructions. Quantification of RNA was also confirmed spectrophotometrically, using NanoDrop ND‐1000 (Thermo Fisher Scientific, Waltham, MA).

### Quantitative Real‐Time PCR (qPCR)

First strand cDNA synthesis was run with 1 µg of total RNA and random primers, using High‐Capacity cDNA Reverse Transcription Kit (Applied Biosystems, Foster City, CA), and following the manufacturer's recommendations. Reactions were prepared by adding 1 µg total RNA, 2 µl of 10× RT Buffer, 0.8 µl of 25× dNTPs mix (100 mM), 2 µl 10× RT primer, 1 µl of Multiscribe Reverse Transcriptase, and nuclease‐free sterile water to 20 µl. Reaction mixtures were incubated at 25 °C for 10 min, 37 °C for 2 h, and 85 °C for 5 min.

The qPCR experiments were run on an Mx3000P Real‐Time System (Stratagene, La Jolla, CA) using SYBR green I with ROX as an internal loading standard. The reaction mixture was 10 µl, comprised of 5 µl of 2× Maxima SYBR Green qPCR Master Mix (Thermo Fisher Scientific), 0.5 µl of cDNA, and 100 nM primers (Integrated DNA Technologies, Coralville, IA). Supplementary Table S1 lists the forward and reverse primers used. Controls included non‐RT controls (using total RNA without reverse transcription to monitor for genomic DNA contamination) and non‐template controls (water template). Specifically, PCR were 10 min at 95 °C, 45 cycles of 15 s at 95 °C, 20 s at 57 °C, and 30 s at 72 °C, 1 min at 95 °C, 30 s at 55 °C, 30 s at 95 °C for At4g24540, *AGAMOUS‐LIKE 24 (AGL24)*; At1g69120, *APETALA1 (AP1)*; At2g46830, *CIRCADIAN CLOCK ASSOCIATED 1 (CCA1)*; At5g15840, *CONSTANS (CO)*; At4g35900, *BZIP TRANSCRIPTION FACTOR FD (FD)*; At1g68050, *FLAVIN‐BINDING KELCH REPEAT F‐BOX 1 (FKF1)*; At5g10140, *FLOWERING LOCUS C (FLC)*; At4g00650, *FRIGIDA (FRI)*; At1g65480, *FLOWERING LOCUS T (FT)*; At1g78440, *GIBBERELLIN 2‐OXIDASE 1 (GA2ox1)*; At4g25420, *GIBBERELLIN 20‐OXIDASE1 (GA20ox1)*; At5g51810, *GIBBERELLIN 20‐OXIDASE2 (GA20ox2)*; At1g22770, *GIGANTEA (GI)*; At4g20400, *JMJC DOMAIN‐CONTAINING HISTONE DEMETHYLASES 14 (JMJ14)*; At5g61850, *LEAFY (LFY)*; At1g01060, *LATE ELONGATED HYPOCOTYL (LHY)*; At3g10480, *NAC TRANSCRIPTION FACTOR 50 (NAC050*); At3g10490, *NAC TRANSCRIPTION FACTOR 52 (NAC052)*; At1g76710, *SET DOMAIN GROUP 26 (SDG26)*; At2g45660, *SUPPRESSOR OF OVEREXPRESSION OF CONSTANS 1 (SOC1)*; At1g62360, *SHOOTMERISTEMLESS (STM)*; At2g22540, *SHORT VEGETATIVE PHASE (SVP)*; At5g03840, *TERMINAL FLOWER1 (TFL1)*; At5g61380, *TIMING OF CAB 1 (TOC1)*; At4g20370, *TWIN SISTER OF FT (TSF)*; At2g17950, *WUSCHEL (WUS)*. Fluorescence was read following each annealing and extension phase. All runs were followed by a melting curve analysis from 55 to 95 °C. The linear range of template concentration to threshold cycle value (Ct value) was determined by preparing a dilution series (0.1–1 µl) using cDNA from three independent RNA extractions analyzed in three technical replicates. Primer efficiencies for all primer pairs were calculated using the standard curve method [Pfaffl, 2001]. Four different reference genes At2g37620, *ACTIN1 (ACT1)*; At5g19510, *ELONGATION FACTOR 1B ALPHA‐SUBUNIT 2 (eEF1Balpha2)*; At1g13440, *CYTOPLASMIC GLYCERALDEHYDE‐3‐PHOSPHATE DEHYDROGENASE (GAPC2)*; and At1g51710, *UBIQUITIN SPECIFIC PROTEASE 6 (UBP6)*, were used to normalize the results of the qPCR. The best of the four genes was selected using Normfinder software (MOMA, Aarhus, Denmark) [Andersen et al., 2004]; the most stable gene was *eEF1Balpha2*. Primers used for qPCR were designed using Primer3 software (Thermo Fisher Scientific) [Rozen and Skaletsky, 2000] and are reported in Supplementary Table S1.

All amplification plots were analyzed with Mx3000P software to obtain Ct values. Relative RNA levels were calibrated and normalized with the level of *eEF1Balpha2* mRNA.

qPCR data are expressed as fold change with respect to the equivalent time‐point in the control.

### Statistical Analyses

In general, the experiments were repeated three times (biological replicates) with at least 15 plants for each experiment. Three technical replicates were run for each biological replicate. Analysis of variance (ANOVA) and Tukey test were used to assess difference between treatments and controls. For generation experiments, at least 15 plants per experiment were used. Data were processed by Kolmogorov–Smirnov test, and Systat 10 (Systat Software, San Jose, CA) was used for univariate and multivariate tests. For all gene expression experiments, at least three samples per treatment group entered the statistical data analysis. Fold change data are expressed as mean values ± standard deviation (SD). Cluster analysis was calculated by using the Systat10 software and by using Euclidean distances with median linkage.

## RESULTS

### NNMF Delays Flowering and Alters Leaf Expansion and Stem Length of *A. thaliana*

**Figure 2:**
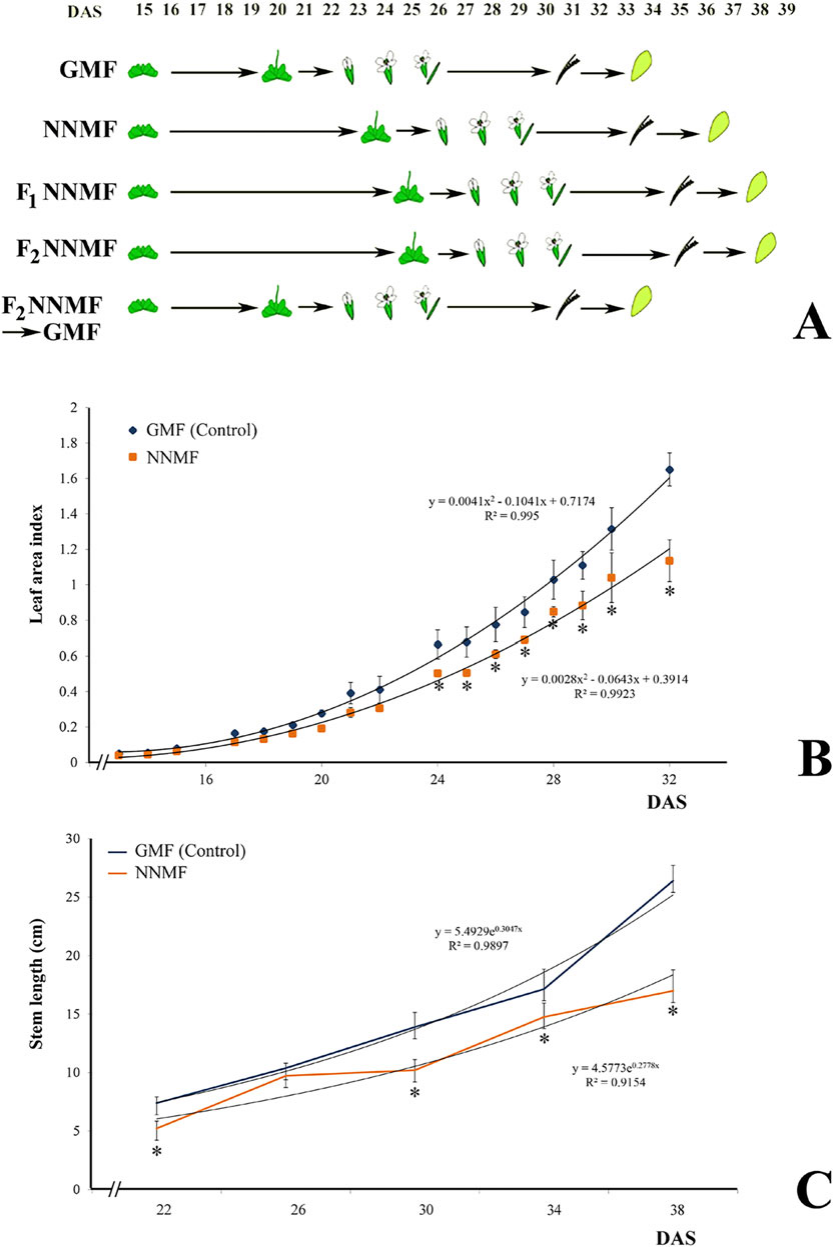
Effect of near‐null magnetic field (NNMF) on *A. thaliana* development and flowering time. (**A**) Phenological phases of *A. thaliana* development and flowering in plant exposed to GMF and to NNMF. (**B**) Leaf area index of plants exposed to normal (GMF) and NNMF. (**C**) Length of flowering stem in controls (GMF) and under NNMF. Metric bars indicate standard deviation; asterisks indicate significant (*P* < 0.05) differences between controls and NNMF. DAS, days after sowing. See also Supplementary Table S2.

*A. thaliana* seed germination in NNMF and GMF did not differ (data not shown); however, exposure of *A. thaliana* to NNMF and long day caused a significant delay in flowering time. NNMF‐exposed plants started flowering about 4 days later with respect to control plants (GMF) and reached full bloom about 5 days later than controls (Fig. 2A, see also Supplementary Table S2 for statistical analyses). We also performed generation experiments to test whether seeds produced under NNMF were affected by further sowing in NNMF. Seeds produced under NNMF (F_1_‐NNMF) germinated regularly but their flowering time was significantly delayed by 6 days with respect to control plants (GMF), and 1 day with respect to parent plants grown in NNMF. A second generation of seeds produced by F_1_‐NNMF, which we indicated as F_2_‐NNMF, did not show any significant difference in flowering time with respect to parent plants F_1_‐NNMF (Fig. 2A, see Supplementary Table S2); however, they still maintained a delay in flowering time with respect to control plants (GMF). When F_2_‐NNMF seeds were sown in GMF, the phenotype and flowering time were found to be the same as plants never exposed to NNMF (Fig. 2A, see Supplementary Table S2). The leaf area index was significantly (*P* < 0.05) lower in plants under NNMF than in control plants (Fig. 2B), and the same results were obtained when the floral stem was measured (Fig. 2C).

### NNMF Alters the Expression of *A. thaliana* Flowering‐Related Genes in Leaves

In order to dissect the effect of NNMF on the transition to flowering, we analyzed gene expression in the leaves.

In the leaves, *A. thaliana* plants under NNMF showed a significant and consistent downregulation of gene expression in early induction times (17–19 days after sowing, DAS) for *CCA1*, *CO*, *FD*, *FKF1*, *FRI*, *FT*, *GA20ox1*, *GA20ox2*, *LFY*, *LHY*, *TOC1*, *TSF*, and *WUS* (Table 1). *AP1*, *GI*, and *STM* were downregulated at later times (19–23 DAS, Table 1), whereas a significant upregulation was found for *FLC* during early floral induction, but the gene was downregulated during later stages of floral development (Table 1). *SDG26* was upregulated at 22 and 28 DAS (Table 1). *A. thaliana TFL1* and *SVP* gene expressions under NNMF were not significantly changed during the early floral induction period and were upregulated during early flowering (23 and 28 DAS, respectively, Table 1). Finally, exposure of *A. thaliana* to NNMF did not cause any significant regulation of *NAC050* in the leaves, whereas it induced a strong downregulation of *GA20ox2* in early floral induction (Table 1).

**Table 1:** Time‐Course Expression of Leaf Genes in *A. thaliana* Exposed to NNMF Conditions

	Days after sowing					
Genes	17	19	21	22	23	28
*AP1*	−1.04 (±0.01)	−**1.71** (±0.11)	−**1.93** (±0.31)	−**3.01** (±0.12)	−**1.40** (±0.04)	1.14 (±0.02)
*CCA1*	−**5.25** (±0.11)	−**1.57** (±0.06)	−1.02 (±0.08)	−**1.65** (±0.03)	−**2.94** (±0.03)	−**1.77** (±0.11)
*CO*	−**2.72** (±0.01)	−**1.74** (±0.13)	1.25 (± 0.4)	−1.13 (±0.20)	1.16 (±0.11)	−**2.70** (±0.04)
*FD*	−**1.40** (±0.03)	−1.23 (±0.09)	−**1.86** (±0.19)	−1.12 (±0.16)	1.18 (±0.09)	−1.14 (±0.18)
*FKF1*	−**1.45** (±0.09)	−**1.67** (±0.02)	−**1.38** (±0.01)	−**1.43** (±0.06)	−1.07 (±0.05)	−1.12 (±0.01)
*FLC*	**1.98** (±0.17)	−1.12 (±0.03)	−1.10 (±0.11)	−**1.75** (±0.11)	−**2.22** (±0.28)	−**3.81** (±0.08)
*FRI*	−**1.80** (±0.09)	−**1.61** (±0.05)	1.27 (±0.57)	−1.32 (±0.04)	**1.71** (±0.07)	−**1.75** (±0.16)
*FT*	−**3.09** (±0.02)	−**2.26** (±0.05)	−1.25 (±0.08)	−1.37 (±0.05)	1.03 (±0.03)	−**2.35** (±0.02)
*GA20ox1*	−**3.11** (±0.28)	−**2.43** (±0.36)	−1.24 (±0.21)	−**2.29** (±0.09)	1.22 (±0.27)	−1.20 (±0.12)
*GA20ox2*	−**5.58** (±0.66)	−**6.81** (±0.34)	1.19 (±0.12)	1.16 (±0.23)	1.38 (±0.18)	−1.02 (±0.11)
*GI*	1.04 (±0.09)	1.07 (±0.11)	1.01 (±0.02)	−**1.32** (±0.50)	−1.04 (±0.04)	**1.51** (±0.05)
*LFY*	−**2.07** (±0.15)	−**1.71** (±0.15)	−**2.15** (±0.23)	1.15 (±0.07)	**1.56** (±0.20)	−**1.66** (±0.27)
*LHY*	−**2.91** (±0.04)	−**1.73** (±0.04)	−1.28 (±0.09)	−**1.77** (±0.05)	−1.04 (±0.05)	−**2.01** (±0.07)
*NAC050*	−1.11 (±0.15)	−1.5 (±0.23)	−1.44 (±0.12)	−1.14 (±0.06)	1.05 (±0.09)	−1.03 (±0.12)
*SDG26*	−1.26 (±0.07)	−1.11 (±0.09)	−1.16 (±0.15)	**2.09** (±0.27)	−1.44 (±0.23)	**3.23** (±0.54)
*STM*	−1.47 (±0.18)	−**2.68** (±0.13)	1.15 (±0.49)	−**1.80** (±0.04)	1.35 (±0.15)	−**1.47** (±0.08)
*SVP*	−1.08 (±0.13)	−1.01 (± 0.1)	−1.31 (±0.20)	1.32 (±0.18)	−1.15 (±0.15)	**1.41** (±0.08)
*TFL1*	−1.52 (±0.18)	−1.11 (±0.16)	−1.33 (±0.13)	1.09 (±0.05)	**2.16** (±0.05)	−1.43 (±0.13)
*TOC1*	−**2.16** (±0.03)	−**1.54** (±0.04)	−**1.39** (±0.09)	−1.13 (±0.12)	−1.23 (±0.02)	−1.13 (±0.03)
*TSF*	−**2.30** (±0.13)	**2.83** (±0.67)	1.14 (±0.50)	−**5.13** (±0.02)	−**6.13** (±0.01)	−**1.70** (±0.24)
*WUS*	−**3.09** (±0.10)	−**2.41** (±0.05)	−1.12 (±0.33)	−1.16 (±0.06)	1.01 (±0.10)	−**1.73** (±0.01)

Boldface numbers indicate significant (*P* < 0.05) difference between treatments and controls.

Values are expressed as fold change (±SD) with respect to control plants growing in GMF conditions. See abbreviation list for gene names in Materials and Methods section.

**Figure 3:**
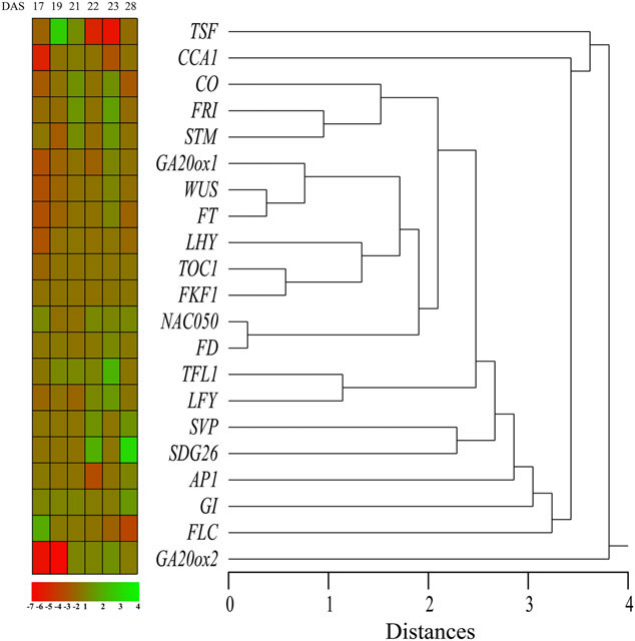
Pattern of expression of genes involved in flowering in *A. thaliana* leaves. Cluster analysis was calculated by using Euclidean distances with median linkage. See text for description. DAS, days after sowing. Different shades of green and red correspond to expression levels reported in the figure color bar.

In order to analyze the pattern of expression of genes in the leaves, a cluster analysis was calculated on the data of Table 1 by using Euclidean distances with median linkage method (Fig. 3). This analysis allowed us to identify possible correlations between genes and to visualize the different patterns of gene expressions with time. We found that *TSF* and *GA20ox2* compose two separate clusters because of late and early downregulation, respectively, whereas *CCA1* and *FLC* form distinct clusters because of their very early (*CCA1*) and late (*FLC*) downregulation. The remaining clusters are made by genes with either late upregulation (*GI*, *SDG26*, and *SVP*), early and late downregulation (*FRI*, *CO*, *WUS*, *FT*, *LHY*), only moderate early downregulation (*TOC1*, *FKF1*), or irregular regulation (all remaining genes) (Fig. 3).

### Reduction of the GMF Alters the Expression of *A. thaliana* Floral Meristem Genes

In the floral meristem of *A. thaliana* plants exposed to NNMF, despite its repressing activity on flowering, *FLC* was significantly downregulated, particularly at 22 DAS (Table 2). During early times of flowering, *LFY*, *SVP*, *SDG26*, and, particularly, *FD* showed a significant upregulation, whereas *AGL24* was significantly downregulated in early times and upregulated during flowering (23, 28 DAS, Table 2). *SOC1* regulation occurred only during late flowering, by showing a downregulation at 28 DAS (Table 2). *LFY* downregulation occurred only after 23 DAS. Upregulation of *AP1* occurred at 22 DAS and was followed by a significant downregulation of the gene between 23 and 28 DAS (Table 2). *GA2ox1* and *GA20ox1* were mildly downregulated in early phase of floral development, whereas a strong downregulation was observed for *GA20ox2* during early flowering (Table 2). Both *NAC050* and *NAC052* were significantly downregulated only at 19 DAS, whereas *JMJ14* did not show any significant regulation (Table 2).

**Table 2:** Time‐Course Expression of Floral Meristem Genes in *A. thaliana* Exposed to NNMF Conditions

	Days after sowing				
Genes	21	22	23	28	30
*AGL24*	−1.06 (±0.08)	−**3.30** (±0.23)	**1.96** (±0.15)	**1.97** (±0.36)	−1.37 (±0.22)
*AP1*	−**1.72** (±0.03)	**1.99** (±0.22)	−**4.75** (±0.01)	−**2.76** (±0.03)	**1.80** (±0.47)
*FD*	**3.41** (±0.55)	−**1.56** (±0.19)	1.39 (±0.02)	−1.16 (±0.13)	−1.02 (±0.03)
*FLC*	−**5.00** (±0.07)	−**14.39** (±0.01)	−**5.54** (±0.03)	−**3.28** (±0.07)	1.75 (±0.18)
*GA2ox1*	−**2.72** (±0.02)	1.03 (±0.05)	−**3.54** (±0.01)	−**3.44** (±0.02)	1.19 (±0.15)
*GA20ox1*	−**1.83** (±0.21)	−**1.92** (±0.25)	−1.39 (±0.23)	1.04 (±0.08)	1.03 (±0.33)
*GA20ox2*	−**23.87** (±5.82)	−**53.65** (±1.23)	−**3.11** (±0.38)	−**47.49** (±5.73)	−1.25 (±0.24)
*JMJ14*	1.10 (±0.32)	−1.17 (±0.21)	1.10 (±0.11)	−1.04 (±0.25)	1.30 (±0.12)
*LFY*	**1.45** (±0.01)	1.27 (±0.14)	−**1.97** (±0.06)	−**2.00** (±0.08)	−1.89 (±0.26)
*NAC050*	1.15 (±0.25)	−**5.79** (±0.96)	1.06 (±0.18)	−**1.95** (±0.35)	−1.42 (±0.22)
*NAC052*	−1.19 (±0.03)	−**4.06** (±0.63)	−1.00 (±0.13)	−**1.60** (±0.10)	−**1.38** (±0.04)
*SDG26*	**1.59** (±0.02)	−**3.31** (±0.75)	1.48 (±0.18)	−1.32 (±0.27)	−**1.61** (±0.32)
*SOC 1*	1.44 (±0.11)	1.69 (±0.20)	−1.15 (±0.06)	−**2.63** (±0.07)	−1.04 (±0.07)
*SVP*	**2.10** (±0.30)	−1.31 (±0.20)	1.16 (±0.25)	−1.13 (±0.19)	−**3.62** (±0.34)

Boldface numbers indicate significant (*P* < 0.05) difference between treatments and controls.

Values are expressed as fold change (±SD) with respect to control plants growing in GMF conditions. See abbreviation list for gene names.

**Figure 4:**
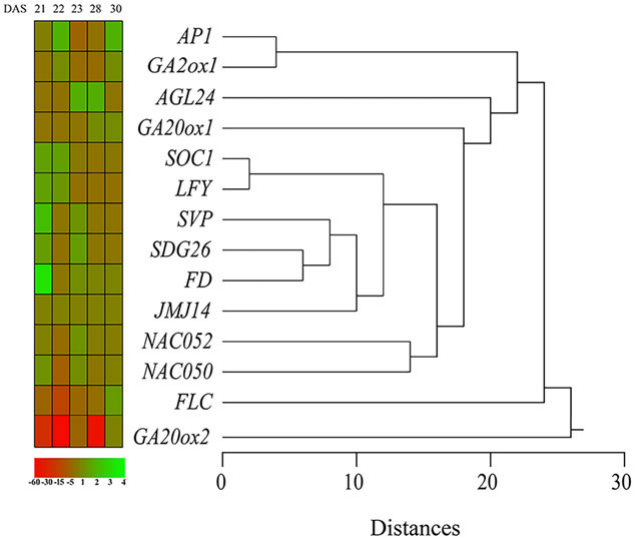
Pattern of expression of genes involved in flowering in *A. thaliana* floral meristem. The cluster analysis was calculated by using Euclidean distances with median linkage. See text for description. DAS, days after sowing. The different shades of green and red correspond to the expression levels reported in the figure color bar.

The cluster analysis calculated on the data of Table 2 by using Euclidean distances with median linkage method (Fig. 4) showed a clear distinction between the pattern of expressions of *GA20ox2* and *FLC* and all other genes. A cluster groups the expression patterns of *AP1* and *GA2ox1*, whereas another cluster groups genes showing early upregulation. The two *NAC* genes (*NAC050* and *NAC052*) are grouped in a cluster because of a similar pattern of expression, whereas the pattern of expression of *AGL24* is separated from the other clusters because of late upregulation (Fig. 4).

## DISCUSSION

In plants, the transition to flowering occurs after floral induction, a period separating vegetative from reproductive development. The timing of floral induction depends on environmental changes and is aimed to maximize reproductive success and seed production. In *A. thaliana* hundreds of genes have been implicated in flowering‐time control [Fornara et al., 2010]. An increasing body of evidence suggests that flowering induction may be delayed by altering the MF of exposed plants. In particular, exposure of *A. thaliana* to NNMF delays flowering time [Xu et al., 2012], but the reasons why this delay occurs are far from clear. For the first time, we showed that this effect was maintained in generation experiments, when plants were constantly grown in NNMF, and normal flowering time was re‐established when plants were grown in GMF. These data indicate that the effect of NNMF occurs in the growing plant, and therefore cannot be due to the conditions at the time the seed was generated. This is strongly suggestive of the presence of a plant magnetoreceptor [Occhipinti et al., 2014] that is able to interfere with the expression of genes that control flowering time [Maffei, 2014]. Furthermore, the observation that germination was not affected by MF variations suggests that in *A. thaliana* the magnetoreceptor must be active in the developing plant (i.e., roots, shoots, or leaves).

The time‐course analysis of leaves and floral meristem genes allowed for evaluation of the different patterns of expression of genes involved in flowering.

### NNMF Downregulates Expression of *A. thaliana* Circadian Clock Genes, Photoperiod, Gibberellin, and Vernalization Pathways

The *A. thaliana* leaf circadian clock is a time‐keeping mechanism that confers diurnal patterns of gene expression and has three interlocked feedback loops. The central loop has the partially redundant transcription factors *CCA1* and *LHY*, which repress transcription of *TOC1*. Although *TOC1* is genetically required for the activation of morning genes, it acts as a repressor and directly regulates the expression of *LHY* and *CCA1. TOC1* also forms a negative feedback loop with *GI* by repressing its expression, and *GI* in turn activates the expression of *TOC1* [Fornara et al., 2010]. In plants under NNMF, we found a significant downregulation of all genes involved in the circadian clock, particularly during the floral induction time. Therefore, we argue that this downregulation might be correlated to the NNMF‐dependent delay in flowering. Flowering of *A. thaliana* is also promoted by photoperiod pathway genes that act in the leaves through a signaling cascade involving *GI* and the transcriptional regulator *CO* [Sawa et al., 2007; Sanchez et al., 2011; Song et al., 2014]. *CO* promotes flowering by initiating transcription of integrator genes *FT* and *TSF* [Hiraoka et al., 2013]. During long days (as those used for exposure under the NNMF), light promotes the interaction between *GI* and *FKF1* proteins, a family of F‐box ubiquitin ligases. These interactions are known to stabilize the F‐box proteins, allowing them to promote the degradation of a set of transcriptional repressors of *CO* [Fornara et al., 2010]. However, our data indicate that these interactions may also suppress *CO* expression. In *A. thaliana* under NNMF, a significant downregulation of *CO*, *FT*, *TSF*, and *FKF1* occurred in the floral induction period, whereas no significant regulation was observed for *GI* (see Fig. 3). Similar effects of NNMF on *CO* and *FT* have been observed in previous works performed in conditions similar to our experiments [Xu et al., 2012].

*GA20ox* enzyme catalyzes several steps in the biosynthesis of GA by oxidizing a number of precursors; furthermore, a reduction of this biosynthetic pathway delays flowering [Brambilla and Fornara, 2013]. NNMF induced a downregulation of *GA20ox2* immediately prior to floral induction, when usually the concentration of bioactive GA (GA_4_) increases at the floral meristem [Fornara et al., 2010].

The transcription factor *LFY* plays a key role in the integration of flowering signals in parallel with *FT* to activate floral meristem identity genes [Abe et al., 2005]. Moreover, in rice *FT* forms a complex with the bZIP transcription factor *FD* and a 14‐3‐3 protein, triggering flowering through the activation of key floral meristem identity genes, such as *AP1* [Taoka et al., 2011]. Under NNMF, expressions of leaf *LFY*, *AP1*, and *FD* were downregulated in the floral induction period. However, *LFY* was upregulated during early flowering; therefore, we suggest that the effect of NNMF on this gene may occur in later stages of plant development. The key floral repressor *TFL1* is an *FT*‐related gene, which maintains the center of the shoot apical meristem (SAM) in a vegetative state by repressing *LFY* and *AP1* [Ratcliffe et al., 1999]. The expression of *AP1* and *TFL1* is antagonistic, as *AP1* represses *TFL1*, and this is likely to be a direct effect as *AP1* directly binds *TFL1* regulatory elements [Kaufmann et al., 2010]. In *A. thaliana* leaves, *TFL1* gene expression under NNMF did not significantly change during floral induction and was upregulated during early flowering (see Fig. 3, 23 DAS).

In the leaves, *SVP* and *FLC* are known to repress the transcription of *FT* [Searle et al., 2006; Jang et al., 2009]. The vernalization pathway activates flowering by silencing *FLC* in response to prolonged exposure to low temperatures [Fornara et al., 2010]. While *SVP* showed only a late and small upregulation in plant exposed to NNMF, a significant upregulation was found for *FLC* during early floral induction, whereas the gene was progressively downregulated during later stages of floral development (see Fig. 3 and Table 1). The regulator gene *FRI* is one of the major determinants of natural variation in flowering time. *FRI* encodes a protein with two coiled‐coil motifs and is required to increase the *FLC* transcript level [Choi et al., 2011]. A slight but significant downregulation of *FRI* was observed in *A. thaliana* exposed to NNMF only in early and very late phase of floral induction (see Fig. 3 and Table 1). This downregulation was associated with the progressive downregulation of *FLC* from early to late stages of development, with the only exception for a significant upregulation of *FRI* at 23 DAS.

In *A. thaliana*, the homeodomain gene *WUS* in the indeterminate shoot apical meristem is essential for maintaining the pool of stem cells, and its downregulation leads to a loss of stem cell activity [Das et al., 2009], whereas the *STM* gene plays an essential role in the establishment and maintenance of indeterminate development of apical and axillary meristems during all phases of plant life [Groot et al., 2005]. Exposure to NNMF downregulated both genes, although *WUS* showed an earlier downregulation with respect to *STM*. The downregulation of *WUS* suggests a reduction of stem cell activity and might be correlated to the flowering delay, while *STM* downregulation suggests a negative effect on the maintenance of vegetative growth, which may justify the observation that NNMF delays but is unable to stop the flowering of *A. thaliana*.

*A. thaliana SDG26* is involved in the activation of flowering, as loss of function of *SDG26* causes a delay in flowering [Berr et al., 2015]. In *A. thaliana* leaves under NNMF, no significant changes were found in *SDG26* regulation before flowering, whereas a significant upregulation was observed during flowering time (see Fig. 3).

The plant‐specific *NAC* proteins form one of the largest transcription factor families in plants [Olsen et al., 2005]. Overexpression of *NAC* transcription factor *NAC050* was found to delay *A. thaliana* flowering time [Ning et al., 2015]; however, despite the evident delay in flowering, exposure of *A. thaliana* to NNMF did not cause any significant regulation of *NAC050* in the leaves.

### NNMF Regulates the Expression of *GA20ox2*, *SVP*, and *FLC* in *A. thaliana* Floral Meristem

Floral induction is necessary to transform the shoot apical meristem from a vegetative meristem to an inflorescence meristem, which forms flowers. This morphological change is associated with dramatic changes in gene expression, including increased expression of the integrator gene *SOC1*, which encodes a MADS‐box transcription factor [Zhao et al., 2014]. In *A. thaliana* floral meristems of plants under NNMF, *SOC1* regulation occurred only during late flowering, by showing a downregulation at 28 DAS (see Fig. 4). *FLC* is also a MADS‐box transcription factor that acts as a potent repressor of flowering and is responsible for much of the variation in flowering time observed in *A. thaliana* [Fornara et al., 2010]. *FLC* and *SVP* work together to repress the expression of *SOC1* [Gregis et al., 2013]. Despite its repressing activity on flowering, *FLC* was significantly downregulated in floral meristems of NNMF‐exposed plants, particularly at 22 DAS (Fig. 4), whereas *SVP* was significantly upregulated at the beginning of the flowering time and downregulated in late flowering. These data indicate that the NNMF‐dependent delayed flowering time might be a consequence of *SVP* upregulation, more than the effect of *FLC*. The interaction of *FT* with *FD* directly promotes the transcription of the MADS‐box factor *AP1* [Brambilla and Fornara, 2013].

A strong and significant downregulation was also observed for *GA20ox2* during the early phases of floral development in NNMF. This regulation could be correlated to the upregulation of *SVP*, which acts either individually or in complex with *FLC* to repress *GA20ox2* expression [Mateos et al., 2015]. On the other hand, *FD* and *AP1* were upregulated in early phases of flower development. These results suggest that upregulation of *FD* at 21 DAS might compensate the strong downregulation of leaf *FT* by inducing *AP1* upregulation at 22 DAS; however, downregulation of *FD* is followed by a significant downregulation of *AP1*. The commitment to flower is ascertained by a direct positive feedback interaction between *LFY* and *AP1* [Valentim et al., 2015]. The transcription factor *LFY* is under direct control of *SOC1* [Valentim et al., 2015] and is involved in the development of a determinate floral meristem [Sablowski, 2007]. As *SOC1*, *LFY* was not regulated during early flowering and was significant downregulated only after 23 DAS, and our cluster analysis confirms the pattern of expression of these two genes.

*AGL24* is one of the MADS‐box genes found to promote flowering [Michaels et al., 2003]. Interestingly, *AGL24* is upregulated during flowering at the same time as the downregulation of *AP1*, *LFY*, and *SOC1*. Upregulated levels of *AGL24* expression correspond to the degree of precocious flowering, and the reduction in *AGL24* expression is related to the degree of late flowering, suggesting that AGL24 is a dosage‐dependent promoter of flowering [Liu et al., 2008]. Since *AGL24* was significantly downregulated in NNMF‐exposed *A. thaliana* in early phases of floral development and was significantly upregulated during flowering, this pattern of expression may indicate that this gene is involved in the later stages of floral development.

Gibberellin is a growth regulator that promotes flowering in *A. thaliana*. *GA20ox* and *GA3ox* can promote the production of active *GA*, whereas *GA2ox* inactivates *GA*, thus regulating its content in plants [Han and Zhu, 2011]. *GA20ox2* was downregulated under NNMF and its pattern of expression was unique, whereas the pattern of *GA2ox1* was similar to *AP1*.

**Figure 5:**
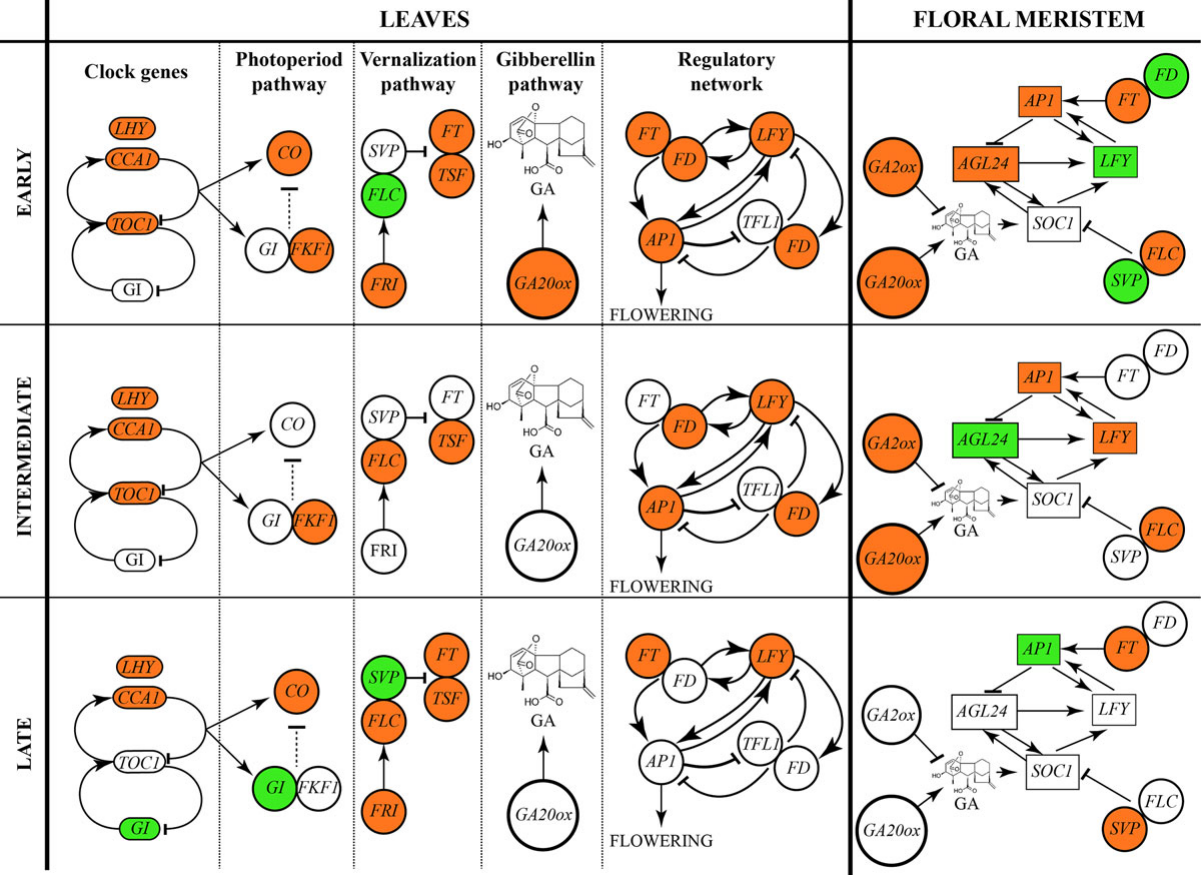
Schematic representation of gene expression patterns in *A. thaliana* leaves and floral meristem under near‐null magnetic field (NNMF). Leaf gene regulation of clock, photoperiod pathway, vernalization pathway, gibberellin pathway, and regulatory network is depicted during early, intermediate, and late stages of flowering according to data of Table 1. An early downregulation of clock, photoperiod, gibberellin, and vernalization pathways is accompanied by a downregulation of *AP1* and *GA20ox*. In the floral meristem (data from Table 2), NNMF determines an early downregulation of the gibberellin pathway, *AGL24* and *AP1*, with a significant upregulation of *LFY*, *FD*, and *SVP*. In both leaves and floral meristem data, upregulation is shown in green, downregulation in light red, and no regulation in white [Fornara et al., 2010; Jaeger et al., 2013; Valentim et al., 2015].

In *A. thaliana*, there are 21 JmjC domain‐containing histone demethylases that have been named JMJ11–JMJ31 [Lu et al., 2008] and H3K4 demethylase JMJ14 is involved in repression of the floral integrator genes *FT* and *SOC1* [Lu et al., 2010]. Recently, JMJ14 was found to be associated with *NAC* transcriptional repressors *NAC050* and *NAC052* [Ning et al., 2015]. In the floral meristem of NNMF‐exposed plants, *JMJ14* did not show any significant regulation, whereas a slight and similar downregulation was found for *NAC050* and *NAC052*. These results indicate that demethylation might not be involved in the delayed transition to flowering caused by exposure to NNMF.

## CONCLUSIONS

The results of this work can be summarized in the scheme of Figure 5 and imply that

- NNMF causes a delay in the transition to flowering due to a combined regulation of leaves and floral meristem genes. An early downregulation of clock, photoperiod, gibberellin, and vernalization pathways is accompanied by a downregulation of *AP1* and *GA20ox*. *FLC* is upregulated by NNMF in early flowering induction. In the floral meristem, the strong downregulation of *FT* and *FLC* in early phases of floral development is accompanied by the downregulation of the gibberellin pathway and upregulation of *FD*, *SVP*, and the transcription factor *LFY*. The common downregulation of *AP1* in both floral meristem and leaves is associated with the delay in flowering.
- In the floral meristem and leaves, the progressive upregulation of *AGL24*, *AP1*, *GI*, and *SVP* from early to late phase of plant development is correlated to the delay of flowering. These events are followed by the progressive reduction of gibberellin pathway downregulation. Our results indicate that NNMF do not prevent flowering, and that variations of the MF are sufficient to modulate specific genes in the early stages of flower induction that are associated with the observed delay.
- The verified delay in the transition to flowering caused by NNMF could be correlated to the observed speciation of Angiosperms after geomagnetic field reversals [Maffei, 2014; Occhipinti et al., 2014; Bertea et al., 2015], which does not exclude a hypothetical influence of GMF magnitude and polarity on plant evolution on a geological time‐scale.
- Since GMF magnitude is not equal everywhere on the Earth's surface, it is possible that changes in GMF in different places could influence plant growth and reproduction.
- However, since the results on gene expression regulation described in this work might not reflect post‐translational modifications that lead to the production of proteins involved in flowering control, further proteomics studies are underway to better assess the role of NNMF on flowering control.
- Finally, experiments with one or more knock‐out mutants of the genes of interest, measuring expression levels in these genotypes, will provide further insight into the nature of triggering events and signal transduction.

Having assessed the downstream events that are associated with a delay in transition to flowering caused by exposure of *A. thaliana* to NNMF, several questions remain unanswered: what is the magnetoreceptor molecule and what is the signaling pathway that induces the gene expression (and repression) reported here?; which point in the life‐cycle of *A. thaliana* is most sensitive to MF perturbations?; and will crops grown in different MF have different productivity? Finally, we note that the expression changes of cryptochrome‐signaling‐related genes *CO* and *FT* shown in this work suggest that the effects of NNMF might be cryptochrome‐related [Xu et al., 2012; Maffei, 2014; Occhipinti et al., 2014]. If a key role for cryptochrome magnetoreception were found in plants, this would make an important link to the mechanism of magnetoreception in avian navigation [Rodgers and Hore, 2009]. Experiments are underway to test this hypothesis and the results will be reported soon.

## Supporting information

Supporting Table S1.

Supporting Table S2.

Supporting Figure S1.
