# Supplementary material for: Reduction of the geomagnetic field delays Arabidopsis thaliana flowering time through downregulation of flowering‐related genes
Source: Bioelectromagnetics. 2018 Apr 30;39(5):361–74. doi: 10.1002/bem.22123 (PMC6032911; doi:10.1002/bem.22123)
Supplement: Supplementary file 1 — Supporting Table S1. [file BEM-39-361-s001.pdf]

Supporting Table S1

Table S1: primers used in quantitative real time PCR experiments

Gene Code	Gene	Forward primer (5'-3')	Reverse primer (5'-3')
**REFERENCE GENES**			
At2g37620	*ACT1*	TGCACTTCCACATGCTATCC	GAGCTGGTTTTGGCTGTCTC
At5g19510	*eEF1Balpha2*	ACTTGTACCAGTTGGTTATGGG	CTGGATGTACTCGTTGTTAGGC
At1g13440	*GAPC2*	TCAGGAACCCTGAGGACATC	CGTTGACACCAACAACGAAC
At1g51710	*UBP6*	GAAAGTGGATTACCCGCTG	CTCTAAGTTTCTGGCGAGGAG-
**FLOWERING GENES**			
At4g24540	*AGL24*	GCGGCTGGAGAAACTACTTG	GCCTCTTTAAGCGTCGTCAG
At1g69120	*AP1*	GCAAGCAATGAGCCCTAAAG	AAGCATGCTGTTTTGCTCCT
At2g46830	*CCA1*	TCAGAAAGGCAAGAGGATGG	ATTCGACCCTCGTCAGACAC
At5g15840	*CO*	ATTCTGCAAACCCACTTGCT	CCTCCTTGGCATCCTTATCA
At4g35900	*FD*	CATCACCTCTCAAACGCTCA	GGAACGAGCTGCAGATTCTC
At1g68050	*FKF1*	CTAAGGTCAGGGGAGGCATAC	ACAGTTGCGAAGGAGAGTGAA

**Gene Code**	**Gene**	**Forward primer (5’-3’)**	**Reverse primer (5’-3’)**
At5g10140	*FLC*	AGCCAAGAAGACCGAACTCA	GGGAGAGTCACCGGAAGATT
At4g00650	*FRI*	ATGCCTGATCGTGGTAAAGG	CGCAGCTAATCCTCCTTCAG
At1g65480	*FT*	CTTGGCAGGCAAACAGTGTA	AGCCACTCTCCCTCTGACAA
At1g78440	*GA2ox1*	GACCAAAACACGGACTCGAT	GGAGGGACAGAGATCCATGA
At4g25420	*GA20ox1*	GCCTTCAAGTCTTTGTGGAA	ATGCAAGTGATTTCCTCTCG
At5g51810	*GA20ox2*	TCGAACGGGATATTCAAGAG	GGTGGTTTCACCACTTTGTC
At1g22770	*GI*	ACGCAGAGACTTCTTCTTGGAC	CAGTTCCTGGGTAGCCTTACAC
At4g20400	*JMJ14*	GTGCTTGACCCAACAAACCT	ACCATTTGCCAGCACTTTTC
At5g61850	*LFY*	GCTCTCCACTGCCTAGACGA	CATGACGACAAGCGATGTTC
At1g01060	*LHY*	GCCATTGGCTCCTAATTTCA	TGTTCCCAACTTGGCTCTCT
At3g10480	*NAC050*	CTCCAATGGACATCGAACCT	GGACGCTCATCTTCTTCTGC
At3g10490	*NAC052*	TCGGACCAACAGAATCATCA	CGCTCCTCTTCTTCCATCTG
At2g45660	*SOC1*	ATCGAGGAGCTGCAACAGAT	GCTTTCATGAGATCCCCACT
At1g76710	*SDG26*	TCGCTCAGAAGCATGTTGAC	TGCTGCTTCCTTCTTCACCT

Gene Code	Gene	Forward primer (5’-3’)	Reverse primer (5’-3’)
At1g62360	*STM*	GTCATCCGAGGAAGAAGTCG	GTAGTGACGGCTCCACCAAT
At2g22540	*SVP*	AGAAGGCCCTTGAAACTGGT	CGCTCGTTCTCTTCCGTTAG
At5g03840	*TFL1*	CAAGGCCAAGCATAGGGATA	GTGCAGCGGTTTCTCTTTGT
At5g61380	*TOC1*	TGATCTCCCAATGGCTAAGG	CATGCGTCTTCTTCTCCACA
At4g20370	*TSF*	CAACCCTCACCAACGAGAAT	ACCGTTTGTCTTCCGAGTTG
At2g17950	*WUS*	ACAACGTAGGTGGAGGATGG	CGCCACCACATTCTTCTTCT
