# Supplementary material for: Reduction of the geomagnetic field delays Arabidopsis thaliana flowering time through downregulation of flowering‐related genes
Source: Bioelectromagnetics. 2018 Apr 30;39(5):361–74. doi: 10.1002/bem.22123 (PMC6032911; doi:10.1002/bem.22123)

Supplementary Table S2

Table S2. Phenological phases of *Arabidopsis thaliana* development and flowering in plant exposed to normal conditions (GMF), NNMF conditions (NNMF), plants produced under NNMF conditions (F_1-NNMF), second generation of plants produced by F_1-NNMF (F_2-NNMF), and plants of F_2-NNMF grown in GMF conditions (F2 NNMF→GMF). Values indicate average days after sowing (± SEM).

**Treatments**	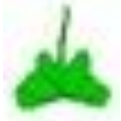	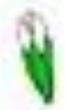	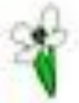	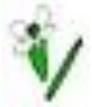	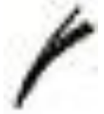	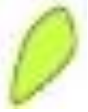
GMF	20 (± 0.18)	23 (± 0.30)	24 (± 0.36)	25 (± 0.56)	31 (± 0.71)	34 (± 0.93)
NNMF	24 (± 0.68)	26 (± 0.49)	27 (± 0.42)	28 (± 0.61)	34 (± 0.72)	37 (± 0.62)
F_1NNMF	25 (± 0.70)	27 (± 0.81)	28 (± 0.81)	30 (± 0.82)	35 (± 0.91)	38 (± 0.59)
F_2NNMF	25 (± 0.83)	27 (± 1.13)	28 (± 0.82)	30 (± 0.89)	35 (± 0.71)	38 (± 1.20)
F_2→GMF	20 (± 0.85)	22 (± 0.56)	24 (± 0.76)	25 (± 0.74)	31 (± 0.64)	33 (± 0.87)

Statistical analyses

Analysis of variance (ANOVA) and the Tukey test were used to assess difference between treatments and controls. For generation experiments, at least 15 plants per experiments were used, data were processed by Kolmogorov-Smirnov test, univariate and multivariate tests by using Systat 10.

**Univariate F Tests**					
**Source**	**SS**	**df**	**MS**	**F**	**P**
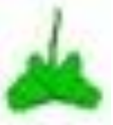	371.267	4	92.817	12.898	<0.001
Error	503.732	70	7.196		
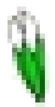	349.231	4	87.308	11.289	<0.001
Error	541.367	70	7.734		
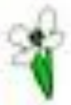	330.749	4	82.687	12.426	<0.001
Error	465.796	70	6.654		
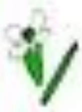	360.284	4	90.071	11.221	<0.001
Error	561.883	70	8.027		
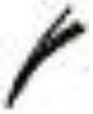	282.702	4	70.676	8.533	<0.001
Error	579.785	70	8.283		

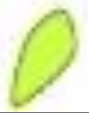	362.638	4	90.66	7.97	<0.001
Error	796.255	70	11.375		

**Multivariate Test Statistics**				
Statistic	**Value**	**F-Statistic**	**df**	**Prob**
Wilks' Lambda	0.291	4.03	24, 227	<0.001
Pillai Trace	0.731	2.533	24, 272	<0.001
Hotelling-Lawley Trace	2.36	6.243	24, 254	<0.001
THETA	S	M	N	Prob
0.7	4	0.5	31.5	<0.001

Kolmogorov-Smirnov Two Sample Test results				
Two-sided probabilities				
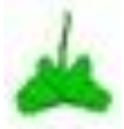				
	F_1NNMF	F_2NNMF	F_2→GMF	GMF
F_2NNMF	0.998			
F_2→NNMF	0.001	0.001		
GMF	<0.001	<0.001	0.006	
NNMF	0.611	0.611	0.001	<0.001

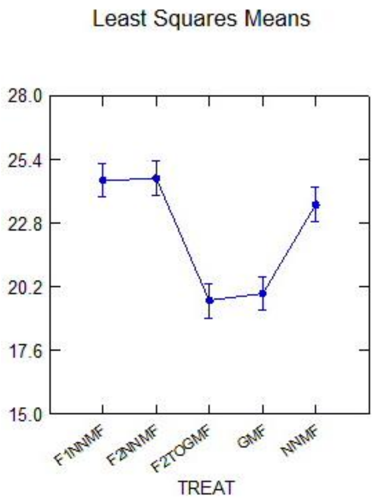

Kolmogorov-Smirnov Two Sample Test results				
Two-sided probabilities				
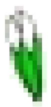				
	F_1NNMF	F_2NNMF	F_2→GMF	GMF
F_2NNMF	0.611			
F_2→NNMF	<0.001	0.001		
GMF	<0.001	0.001	0.160	
NNMF	0.035	0.341	0.001	<0.001

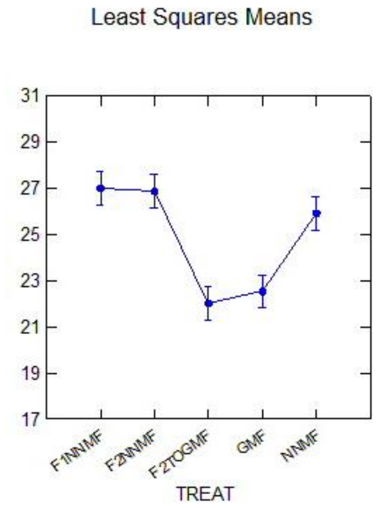

Kolmogorov-Smirnov Two Sample Test results				
Two-sided probabilities				
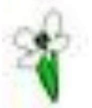				
	F_1NNMF	F_2NNMF	$F_2\rightarrow$GMF	GMF
F_2NNMF	0.998			
$F_2\rightarrow$NNMF	0.022	0.022		
GMF	<0.001	<0.001	0.341	
NNMF	0.611	0.611	0.006	<0.001

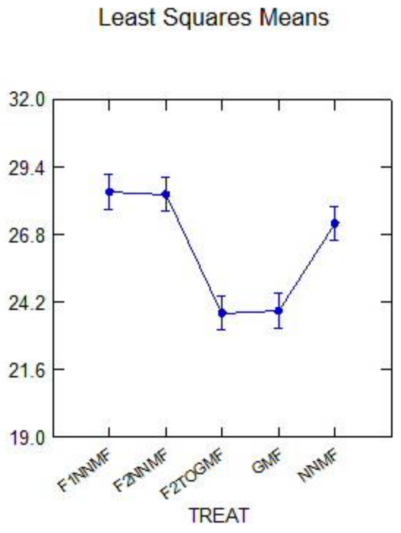

Kolmogorov-Smirnov Two Sample Test results				
Two-sided probabilities				
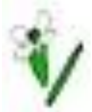				
	F_1NNMF	F_2NNMF	$F_2\rightarrow$GMF	GMF
F_2NNMF	0.998			
$F_2\rightarrow$NNMF	0.001	0.001		
GMF	<0.001	0.001	0.894	
NNMF	0.341	0.160	0.065	0.022

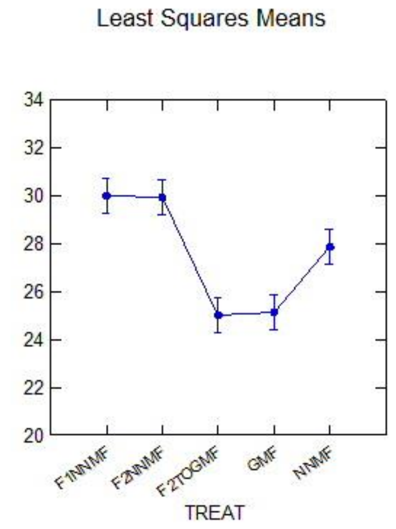

Kolmogorov-Smirnov Two Sample Test results				
Two-sided probabilities				
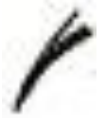				
	F_1NNMF	F_2NNMF	F_2TOGMF	GMF
F_2NNMF	0.894			
$F_2\rightarrow$NNMF	0.006	<0.001		
GMF	0.065	0.006	0.894	
NNMF	0.341	0.341	0.160	0.065

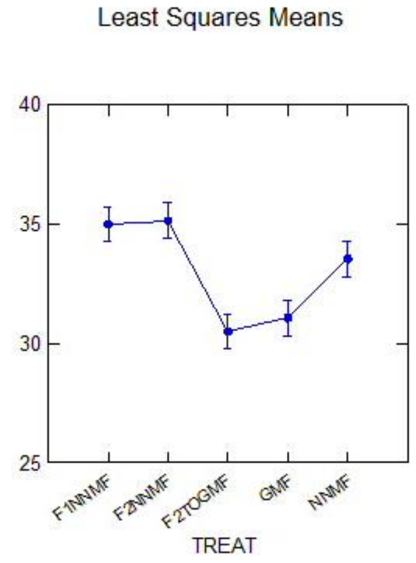

Kolmogorov-Smirnov Two Sample Test results				
Two-sided probabilities				
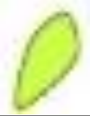				
	F_1NNMF	F_2NNMF	$F_2 \rightarrow$GMF	GMF
F_2NNMF	0.894			
$F_2 \rightarrow$NNMF	0.006	0.006		
GMF	0.006	0.022	0.611	
NNMF	0.611	0.341	0.065	0.065

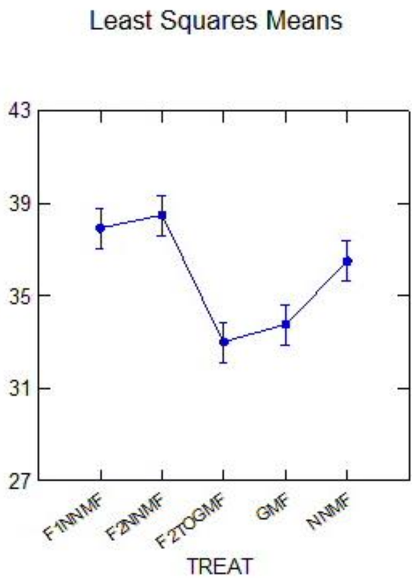

Supplement: Supplementary file 2 — Supporting Table S2. [file BEM-39-361-s002.pdf]
