# Supplementary material for: Reduction of the geomagnetic field delays Arabidopsis thaliana flowering time through downregulation of flowering‐related genes
Source: Bioelectromagnetics. 2018 Apr 30;39(5):361–74. doi: 10.1002/bem.22123 (PMC6032911; doi:10.1002/bem.22123)
Supplement: Supplementary file 3 — Supporting Figure S1. [file BEM-39-361-s003.pdf]

US/UK World Magnetic Model - Epoch 2015.0
Main Field Inclination (I)

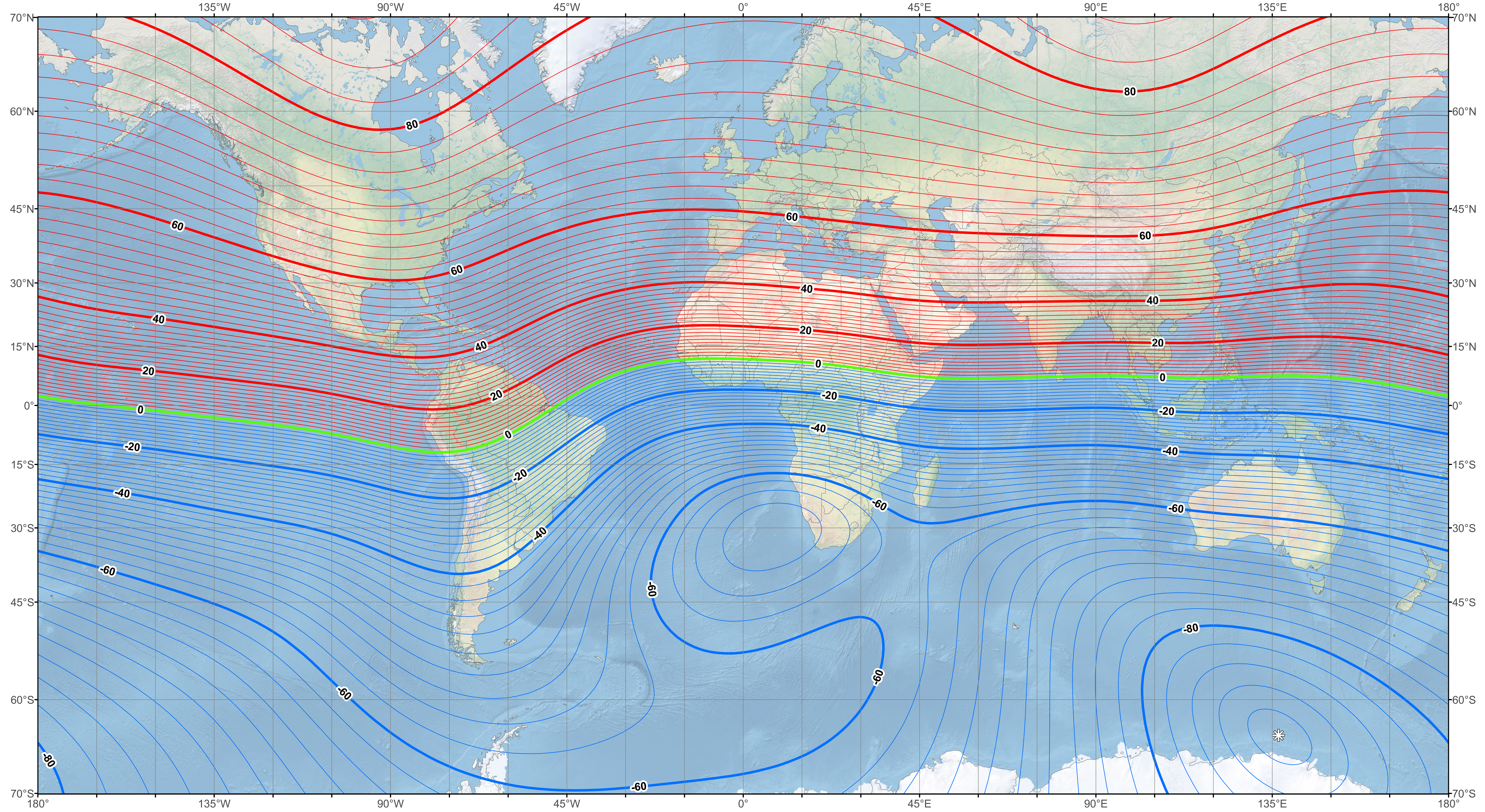

Main field inclination (I)
Contour interval: 2 degrees, red contours positive (down); blue negative (up); green zero line.
Mercator Projection.
: Position of dip poles

Map developed by NOAA/NGDC & CIRES
http://ngdc.noaa.gov/geomag/WMM
Map reviewed by NGA and BGS
Published December 2014
